# Measuring the elasticity of liquid–liquid phase separation droplets with biomembrane force probe

**DOI:** 10.52601/bpr.2022.210038

**Published:** 2022-04-30

**Authors:** Min Sun, Hui Chen, Qinghua Ji, Jianhui Xiao, Yanzhe Hou, Jizhong Lou

**Affiliations:** 1 Key Laboratory of RNA Biology, CAS Center for Excellence in Biomacromolecules, Institute of Biophysics, Chinese Academy of Sciences, Beijing 100101, China; 2 University of Chinese Academy of Sciences, Beijing 100049, China; 3 School of Basic Medical Pharmacology, Lanzhou University, Lanzhou 730000, China

**Keywords:** Phase separation, Force spectroscopy, Young’s modulus

## Abstract

Numerous biomacromolecules undergo liquid–liquid phase separation (LLPS) inside living cells and LLPS plays important roles in their functions. The droplets formed by LLPS molecules are complex fluids and their behavior follows fluid mechanics, thus studies on rheological and material properties are required to gain full insight into the biophysical mechanism of these droplets. Biophysical force spectroscopy techniques are particularly useful in this aspect. Indeed, atomic force microscopy and optical tweezers have been used to quantify the elasticity and the viscoelasticity of LLPS droplets. The Biomembrane Force Probe (BFP) is a single-molecule technique designed to investigate liquid-like objects and is more suitable to quantify the material properties of LLPS droplets, but its usage on LLPS droplets is not yet described. Here we present an experimental protocol to measure the Young’s modulus of LLPS droplets using BFP, we believe that the application of BFP on phase separation studies can be expanded and will be very helpful in deciphering the underlying principles of LLPS.

## INTRODUCTION

Phase separation of biomacromolecules has been extensively studied in cell biology. Biomacromolecules interact with each other to form condensates (or droplets if in three dimensions) with different material properties and play important roles in chromatin organization, gene expression and signal transduction (Banani* et al.* 2017). Phase separated condensates could be classified as liquid-like, gel-like and solid-like depend on their molecular dynamics quantified by FRAP analysis (Taylor* et al.* 2019; Zhang* et al.* 2020). Plenty of studies focus on the relationship between condensates’ biophysical properties and physiological functions (Bracha* et al.* 2018; Elbaum-Garfinkle and Brangwynne 2015; Murakami* et al.* 2015; Shin* et al.* 2018). A lot of physiological conditions could mediate the transition of condensates’ material properties, and many aberrant transitions have been proved to be disease-relevant.

As complex fluids, droplets formed via liquid-liquid phase separation (LLPS) of biomacromolecules need to be described by fluid mechanics, and appropriate quantification on their material properties is required for a comprehensive description. To better characterize the material properties of condensates, various methods have been developed to study the viscoelasticity and surface tension of these phase-separated condensates. Single-molecule force spectroscopy techniques are very helpful. Atomic force microscopy (AFM) studies indicated that the viscoelasticity of distinct condensate not only reflects its material properties but also molecular dynamics within the condensate (Zeng* et al.* 2018). Optical tweezers (OT) measurements indicated that droplets formed by P-granule protein PGL-3 are predominantly viscous but also exhibit elastic behavior (Jawerth* et al.* 2018), and the studies further reveal that the droplets behave as aging Maxwell fluid (Jawerth* et al.* 2020). Previous studies also show contradictory results in the size dependency of elastic modulus of condensates (Evangelopoulos* et al.* 2012; Poling-Skutvik* et al.* 2020; Zeng* et al.* 2018).

The Young’s modulus of an LLPS droplet describes the tensile elasticity of the droplet, that is, the ability to change in length or dimension when stretched or compressed. It had been found to be important for the biological function of the condensates. Studies revealed that the participation of more postsynaptic density (PSD) scaffold proteins increases the Young’s modulus of the PSD LLPS condensates, which may stabilize the condensates, facilitating their biological functions such as clustering glutamate receptors, enriching synaptic enzymes and promoting actin bundle formation (Zeng* et al.* 2018). It was also evidenced that nuclear condensates favor growth in softer, lower-density regions of the genome as a result of the deformation energy of the elastic condensates, giving rise to genomic rearrangements and promoting active gene expression in particular genomic regions (Shin* et al.* 2018). These studies highlight the importance to quantify the elasticity of the LLPS droplets, especially the Young’s modulus.

Here we present a protocol for Young’s modulus measurement using a home-built biomembrane force probe (BFP), and the size of each droplet could also be measured at the same time. BFP is much easier to control than an OT, hundreds of measurements could be performed for many individual droplets. Moreover, the heat effect from the strong laser used in OT could potentially damage the droplet structure and alter its material property. While for AFM measurements, the droplets may spread out on the substrate surface and the mechanical properties of the droplet may also be altered. The BFP is specifically designed to measure liquid-like objects and is much useful for the measurement of LLPS droplets, it could provide statistically more accurate quantification of the Young’s modulus of the droplets.

## PRINCIPLES

### The biomembrane force probe

The BFP was first introduced by Evans *et al*. in 1995 as a sensitive force technique to probe molecular adhesion and structural linkage at biological interfaces (Evans* et al.* 1995). Similar to other single-molecule force spectroscopy techniques, the BFP can measure the strength, lifetime and force to rupture a biological molecule or interacting molecule pair under force loading. Specifically, the BFP uses a pressurized red blood cell (RBC) as an ultrasensitive force transducer. With adjustable RBC spring constant ranging from 0.1–1 pN/nm, the BFP can detect force ranging from 1–1000 pN which makes it a powerful tool to measure the molecular forces, such as the ligand–receptor interaction and intramolecular bonds (Chen* et al.* 2015; Ju 2019; Wu* et al.* 2019). More importantly, the BFP was operated by micropipettes aspiration, making it more suitable for live cell or liquid droplet measurements.

**Figure 1:**
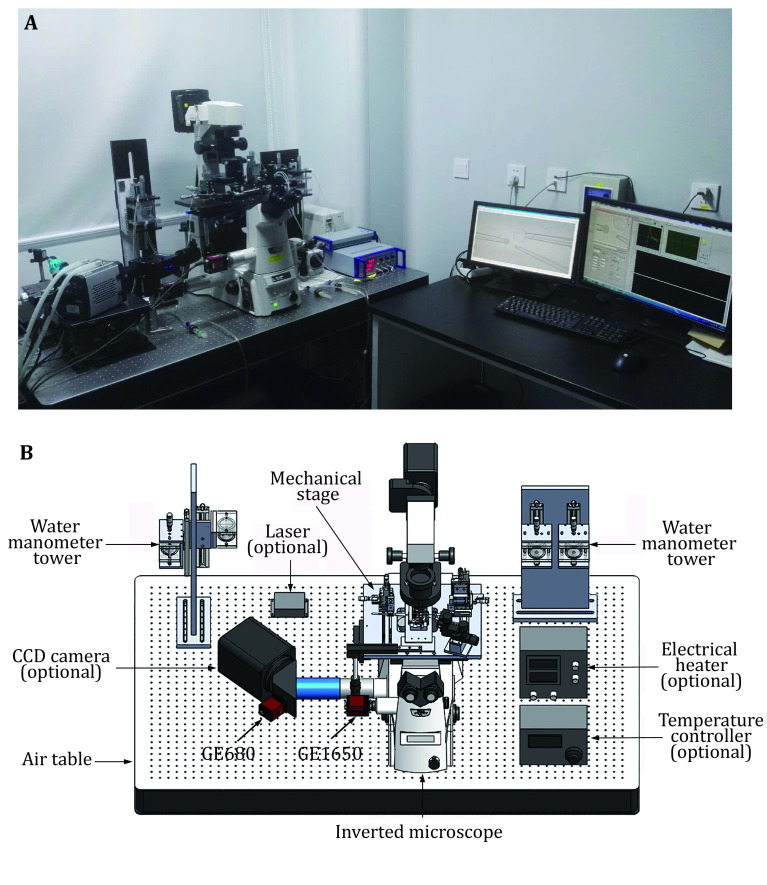
Biomembrane force probe instrumentation. **A** Picture of a home-made BFP. It is built based on an inverted microscope. **B** Schematic of the necessary parts required to build a BFP. Optional parts are indicated. The experimental chamber and the micromanipulators are positioned on the mechanical stage

A functional BFP instrument includes a hardware system and a software system (Fig. 1). To reduce mechanical fluctuation, the BFP is generally built on an air table. The hardware consists of optical and mechanical modules controlled by computers or controllers. The optical module includes an inverted biological microscope, laser and light sources, and at least two cameras. The inverted microscope contains a high magnification objective lens (for example, 40X/NA0.75) and several video tubes. A mercury lamp, which is able to provide illumination on the experimental chamber, is used as the light source. Two cameras, a normal speed one and a high speed one, are required and can be mounted on the microscope via video tubes (Fig. 1A and 1B). The normal speed camera is used to monitor the experiment process and the high speed camera is used to trace the deformation of the RBC to quantify the force applied (Fig. 1B). Fluorescence measurement is also able to be integrated into the BFP system, where additional lasers and cameras are required (Fig. 1B) (Chen* et al.* 2015).

**Figure 2:**
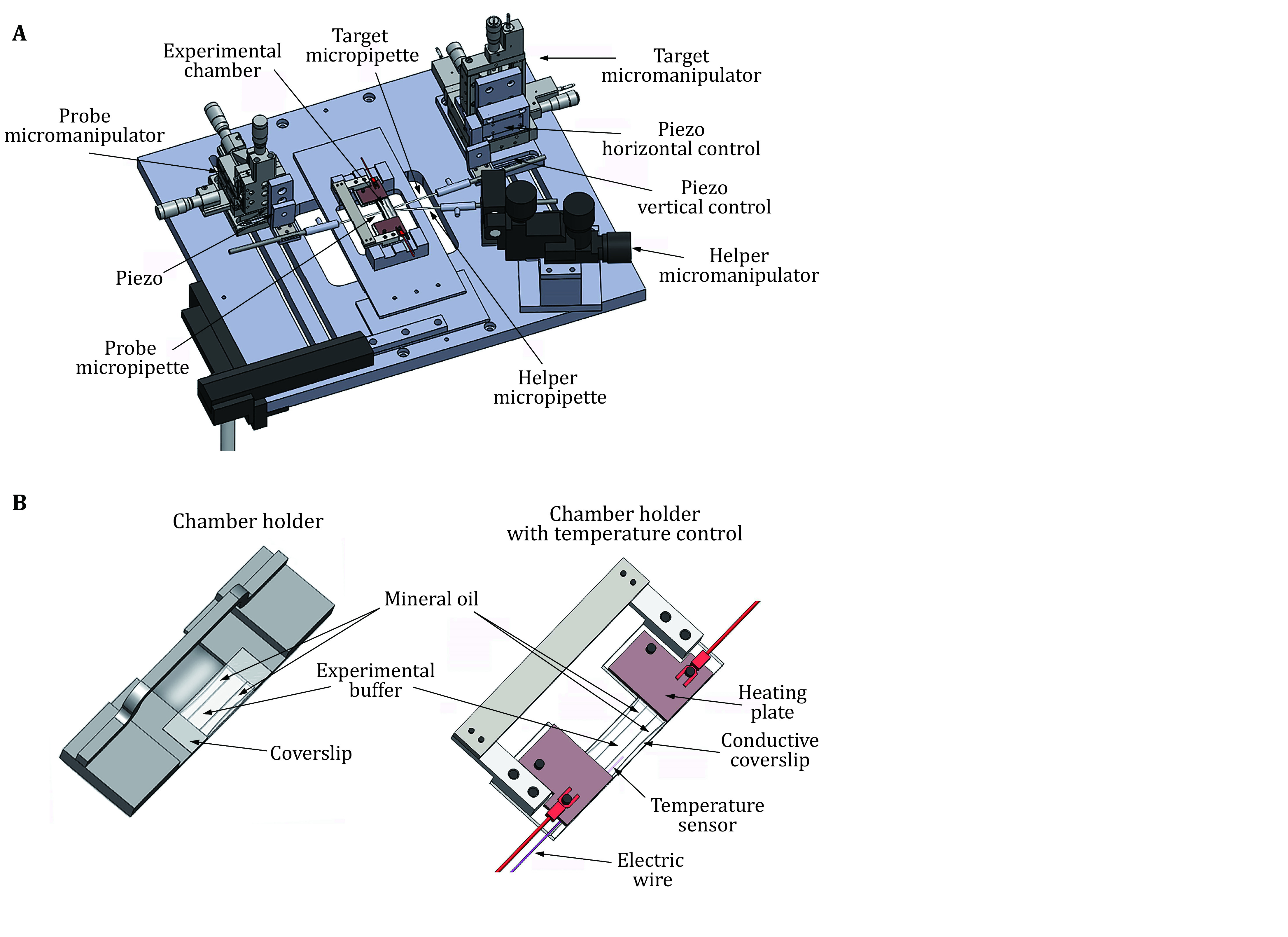
Setup of the mechanical stage and the experimental chamber. **A** The mechanical stage. Three micromanipulators are installed in the mechanical stage, controlling the probe, target and helper micropipettes respectively. Piezoes are mounted on the target micromanipulator which can provide movement control in nanometer resolution. The chamber holder is placed on the stage at the position right above the objective lens. **B** Schematics of the chamber holder. The experimental chamber was formed by the facing edges of the opposite metal plates of the holder, and the coverslips glued on the top and the bottom of the metal plates, leaving the other two sides open to allow the insertion of the micropipettes. In the experiment, the experimental buffer, the RBC, beads, and the cell or droplet samples are injected into the chamber, and the two open sides of the chamber are sealed with mineral oil. The mineral oil can prevent the experimental buffer from evaporation. If temperature control is required in the experiment, a revised version of the chamber holder (right) can be manufactured

The mechanical module includes an air table, a mechanical stage with three micromanipulators and three water manometer towers. The air table supports the main parts of the system and isolates the environmental mechanical vibrations to reduce measurement fluctuation. The mechanical stage supports the micromanipulators and the experimental chamber, and can move on the horizontal axis to adjust the position of the experimental chamber for better observation. In BFP, three micromanipulators are required to hold and position three micropipettes which are denoted as the probe (aspirating an RBC), the target (aspirating a target cell or droplet), and the helper (aspirating a bead and attaching it to the RBC) micropipettes (Fig. 2A). Each micromanipulator is fixed on the mechanical stage and provides a micropipette with 3D localization at sub-micrometer resolution. After mounting with the micropipette holder, the attached micropipettes point to the middle of the experimental chamber, the tips of the micropipettes can reach each other after micromanipulator adjustment. At least one piezo actuator should be mounted onto the target micromanipulator, which allows sub-nanometer precision movement along the axial direction to enable attachment and detachment of the target to the probe. Each micropipette will be connected to a water manometer tower, such that the water pressure inside the micropipette can be adjusted. The manometer connected to the probe micropipette contains a pressure sensor to measure the pressure differences on this micropipette, which will be used to determine the spring constant of the probe (RBC).

The mechanical stage contains a groove to mount the chamber holder (Fig. 2A). The chamber holder is made with heavy metal (such as steel) to reduce the vibration of the chamber (Fig. 2B). The chamber is formed by two coverslips glued onto the top and bottom surface of the opposite chamber holder plate. The thickness of the plate defines the vertical length of the chamber and the remaining two opposite sides of the chamber are open to allow micropipettes insertion. A new chamber is assembled for each experiment, and it will be discarded after the experiment and the chamber holder should be thoroughly cleaned. The experimental buffer is injected into the chamber with a syringe or a pipette in the middle of the chamber with the edges of the two open sides sealed with mineral oil (Fig. 2B). The mineral oil seals the open sides of the chamber and keeps the experimental solution from evaporation. A chamber holder with temperature control could be designed for experiments performed at a desired temperature higher than the room temperature (Fig. 2B).

This BFP hardware is controlled by a user-written LabVIEW program. The software program includes an image-processing module and a piezo-controlling module. The image-processing module grabs and analyzes the images captured by the cameras. The piezo-controlling module controls the movement of piezo(s) mounted on the target micromanipulator.

**Figure 3:**
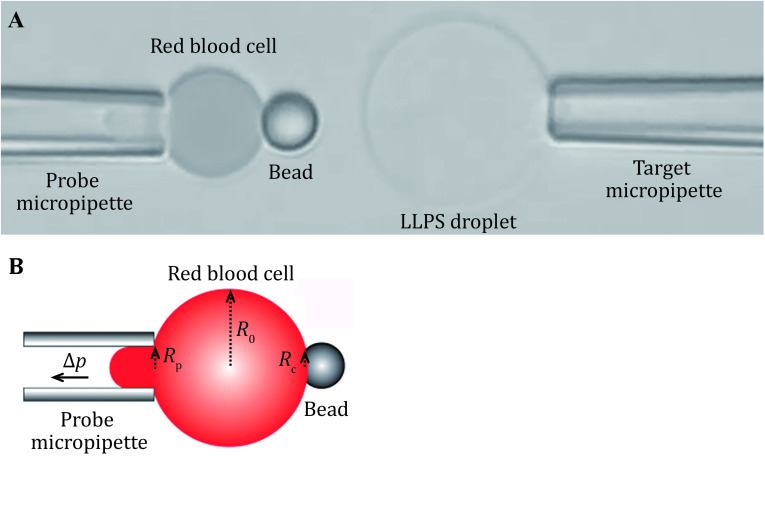
The micropipettes system for target and probe aspiration. **A** Image of the whole view field in the BFP experiment. The probe micropipette first aspirates a swollen biotinylated RBC, then an SA-bead was attached at the opposite side of the RBC by the helper micropipette. A cell or liquid droplet is aspirated by the target micropipette whose movement is controlled by a Piezo actuator. **B** Measureing the spring constant of the RBC. A camera images the whole view field including the micropipette, the RBC and the bead. The radii of the probe micropipette tip (*R*_p_), the RBC (*R*_0_) and the binding interface of the RBC/SA-bead (*R*_c_) can then be measured. Together with the pressure differences Δ*p* detected by the pressure sensor, the spring constant *k*_*p*_ of the RBC system could be calculated

During the BFP experiment, experiment samples (RBCs, beads, cells or droplets) are injected into the experimental buffer solution at different chamber locations, generally, they will settle at the surface of the bottom coverslip. These samples will be aspirated by the corresponding micropipette under negative pressure from the water manometer tower. The probe micropipette aspirates an RBC, the helper micropipette aspirates a bead and is manipulated to place the bead onto the RBC at the opposite side of the probe micropipette aspiration (Fig. 3A). Because the bead is coated with streptavidin (SA) and the swollen RBC is biotinylated, they can interact with each other and form a stable contact surface. After the successful attachment of the bead with the aspirated RBC, the helper micropipette could be removed or moved away from the experimental view field. The target micropipette aspirates a cell or a droplet (Fig. 3A).

The normal speed camera monitors a larger view field including the probe and the target (that is, a portion of the micropipettes, RBC, bead and target cell), which helps to oversee the experimental process.

The control and imaging of the high speed camera are integrated into the operation program. Before force measurements, the high speed camera takes a picture of the whole view field. From this picture, the inner radius at the tip of the probe micropipette $ {R}_{\mathrm{p}} $, the radius of the RBC $ {R}_{0} $ and the radius of the contact interface between the RBC and SA bead$ {R}_{\mathrm{c}} $ can be measured (Fig. 3B). The spring constant $ {k}_{p} $ of the RBC can be calculated based on the equation:

1 $ {k}_{p}=\dfrac{\pi {R}_{\mathrm{p}}\Delta p}{\left(1-\dfrac{{R}_{\mathrm{p}}}{{R}_{0}}\right)\mathrm{l}\mathrm{n} \left(\dfrac{4{R_{0}}^{2}}{{R}_{\mathrm{p}}{R}_{\mathrm{c}}}\right)} \;\;, $

where Δ*p* is the aspiration pressure applied on the probe micropipette which can be measured by the pressure sensor.

**Figure 4:**
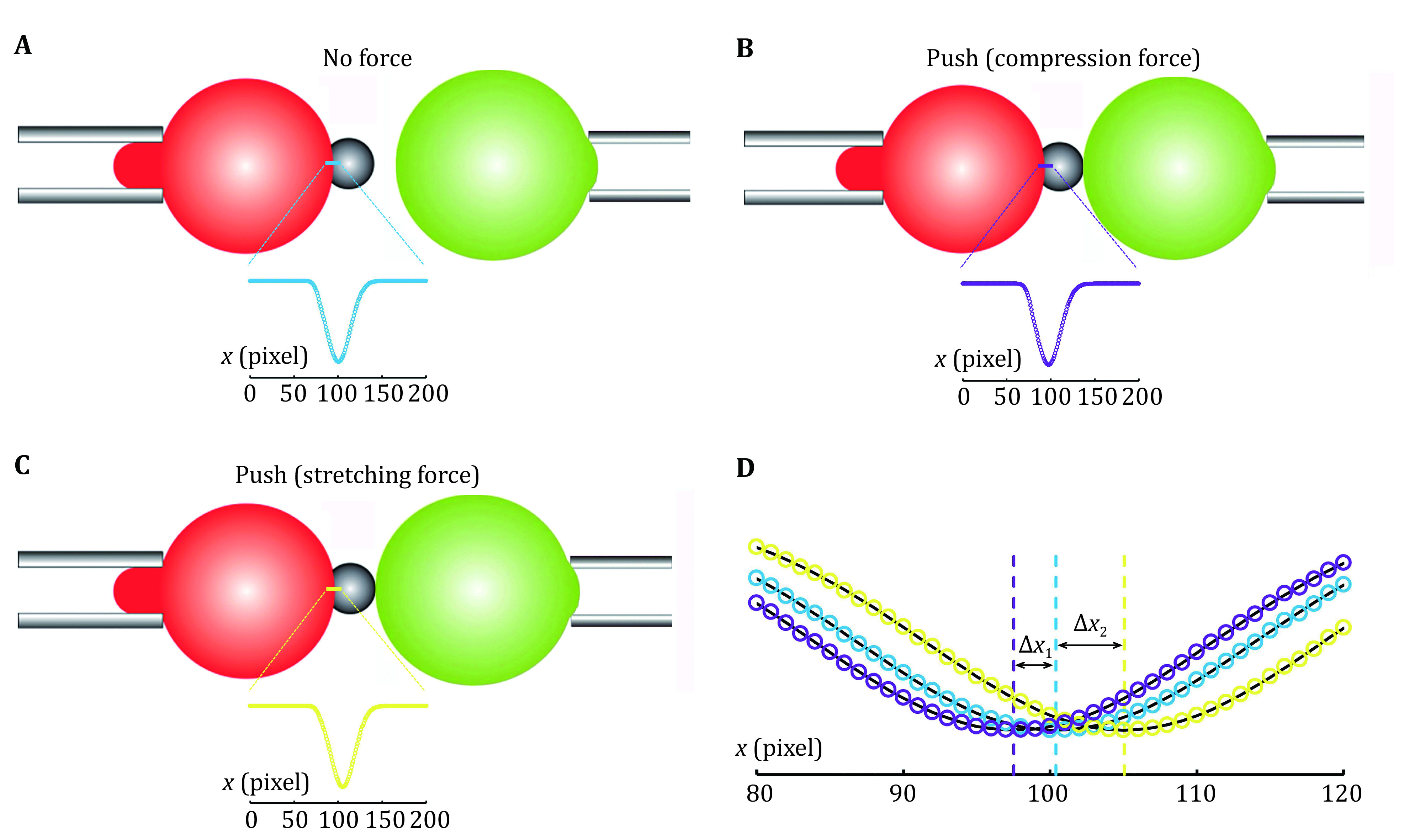
Measuring RBC deformation in BFP. **A–C** In BFP, a high speed camera images a thin line at the boundary between RBC and the SA-bead (cyan in Panel A, purple in Panel B, and yellow in Panel C). The intensity profiles of the images (insets) are captured and analyzed in the unit of the pixel of the camera. The profile of no force condition (A) serves as a reference for RBC conformation, and that for pushing (B) or pulling (C) can be compared with the reference. **D** Gaussian distribution fitting of the intensity profile of each image indicates the deformation of the RBC in pixel (Δ*X*_1_ and Δ*X*_2_), which can be converted to distance change (Δ*d*) by multiplying the resolution of each pixel

During the force measurements, the high speed camera images a thin line which crosses the center of the RBC/SA-bead contact interface (Fig. 4). The high speed camera can image this thin line at a very high speed (more than 1,500 frames per second). Each image will be captured by the software program, and the light intensity along the line can be obtained and analyzed, the RBC/SA-bead boundary appears darker with different light intensity than the location away from the boundary (Fig. 4). When the cell or droplet aspirated by the target micropipette is far away and stays still, the probe side is at the resting state, that is, the system experiences no force (Fig. 4A). When the target micropipette is controlled to move closer, the aspirated cell or droplets pushes the bead at the probe side, compression force is generated and transmitted to the RBC, resulting in its deformation, and the RBC/SA-bead boundary shifts leftward (Fig. 4B). If the cell or droplet can form interaction with the bead, the stretching force can be generated and deform the RBC to make RBC/SA-bead boundary shifts rightward (Fig. 4C). The deformations of the RBC can be monitored by the light intensity analyzed from the high speed camera images. After fitting the light intensity curves with Gaussian distribution, the peak position of the Gaussian distribution provides the position of the RBC/SA-bead boundary (Fig. 4D). The deformation of the RBC in pixel (Δ*X*) can be obtained using the peak position of the force free condition as reference. Each pixel corresponds to the resolution of the high speed camera, which is a fixed value for a BFP and is governed by the magnifications of the objective lens, the video tube lens and the camera itself, generally in several tens of nanometers. The deformation of the RBC (Δ*d*) can be obtained by multiplying the resolution of the pixel with the pixels changed (Δ*X*). Then the compression or stretching force can be calculated by

2 $ F={k}_{p}\times \Delta d \;. $

By fitting the images acquired by the high speed camera in time series, the time dependent force curve can be obtained.

The BFP is beneficial to study the mechanical properties of soft spherical objects, especially live cells or droplets. It had been successfully applied to study the interaction kinetics of cell surface receptors with their ligands, including integrins (Chen* et al.* 2010, 2012, 2019; Ju* et al.* 2018), platelet GPIb (Ju* et al.* 2015a, b), and T-cell receptors (Liu* et al.* 2014; Sibener* et al.* 2018; Wu* et al.* 2019), these studies provided new information to understand the biological functions of these systems. New methods or designs to improve the accuracy, efficiency and application were also developed. A thermal fluctuation assay was proposed to determine receptor–ligand association/dissociation kinetics by monitoring abrupt decrease/resumption in thermal fluctuations of BFP curves (Chen* et al.* 2008). A fluorescence BFP was designed to observe single-molecule force spectroscopy and the resulting signals inside a living cell at the same time (Chen* et al.* 2015; Liu* et al.* 2014). A dual BFP system was constructed which enables single-cell mechanical analysis of signal crosstalk between multiple molecular species (Ju* et al.* 2017). Recently, an ultra-stable BFP system for measuring biological interactions with slow dissociation kinetics was established, improving the accuracy of BFP measurement (An* et al.* 2020). These improvements or modifications broaden the potential applications of BFP.

Besides energy profiles and association/dissociation measurements, the BFP can be used to measure the elasticity of samples, such as the droplets. The force curve was generated when a droplet was compressing the RBC in controlled pressing parameters, so that we can obtain the Young’s modulus of the droplet from the curve to reflect the elasticity of the droplet which provides the information of the molecular interaction within the droplets. Although the overall procedure to perform Young’s modulus measurement of the LLPS droplet with BFP was quite similar to the conventional applications of BFP for quantification of receptor/ligand interactions, there were some differences. Firstly, the probe beads did not need to be functionalized with ligands or biomolecules. Secondly, the formation of most LLPS droplets was concentration dependent, thus the buffer condition for measuring droplet elasticity should be carefully adjusted to maintain the stability of the droplets.

### Elasticity measurement with BFP

**Figure 5:**
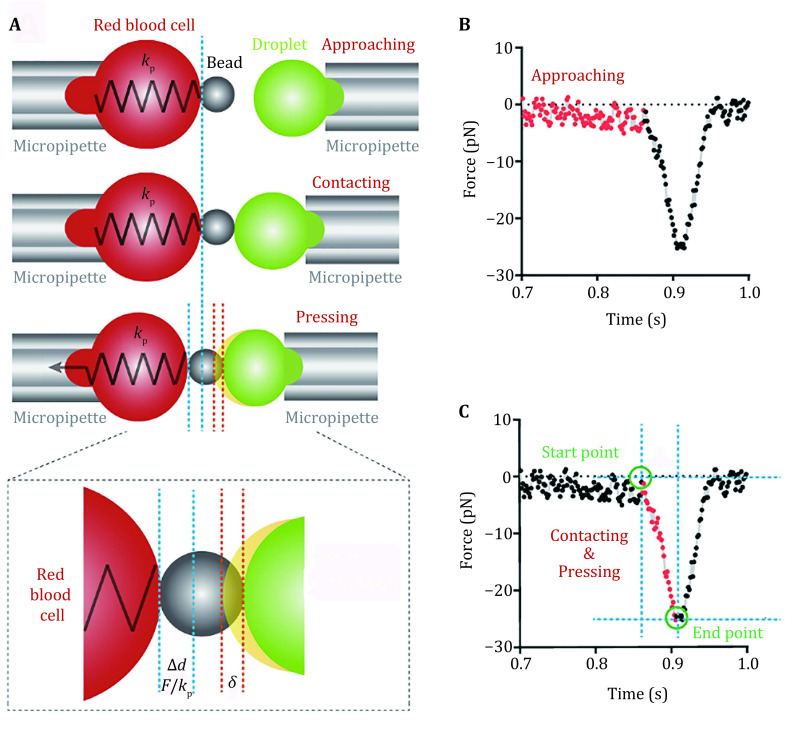
Measuring the Young’s modulus of LLPS droplets. **A** A droplet is aspirated by the target micropipette. An experimental cycle of the droplet approaching, pressing and retracting from the bead on the probe. **B** A typical force curve generated from the experimental cycle shown in Panel A, the approaching phase is shown in red. **C** In the pressing process (red), the force and time of starting and ending points can be used to calculate the Young’s modulus$ E $

In BFP, an LLPS droplet is aspirated by the target micropipette and is controlled to approach the probe (Fig. 5A). Before contacting, no net force exists, the measured force fluctuates around the ground level. Right after target/probe contacting, compression force will be generated and increase as the target continues moving leftward to press the probe (Fig. 5B). The force can be measured by monitoring the deformation of the RBC, and the deformation of the droplet (indentation depth *δ*) can be obtained by subtracting RBC deformation from the piezo movement (Fig. 5A). After a successful pressing, the target will move backward to separate from the probe and the force will also decrease to ground level (Fig. 5B), which completes a measurement cycle. To measure the elasticity of a droplet sample accurately, hundreds of such measurement cycles should be performed.

Different models have been developed to derive mechanical properties of samples from the force curves such as that shown in Fig. 5B. Among these models, the Hertz model was widely used in calculating the mechanical properties in an assumed condition where the sample was continuous, smooth and the deformation was relatively small (Kuznetsova* et al.* 2007). The LLPS droplets fit the condition well. Thus, for BFP measured force curves, the Hertz model can be used to derive the Young’s modulus of the droplets.

When the force probe was spherical, the Hertz model was described as:

3 $ F=\frac{4}{3}\frac{E}{1-{v}^{2}}\sqrt{R}{\delta }^{\tfrac{3}{2}} , \;\;$

where the *F* denotes the force applying on the droplet, *E* is the Young’s modulus, *v* is Poisson’s ratio and is typically valued as 0.5 for biomaterials. *R* is the radius of the probe and $ \delta $ denotes the indentation depth.

There are two main methods to obtain the Young’s modulus according to the Hertz model (Kuznetsova* et al.* 2007): slope fitting method and two-point method. Here we used the two-point method which includes the information of contact starting point and ending point to acquire Young’s modulus (Fig. 5C).

4 $ F=\left|{F}_{\mathrm{s}\mathrm{t}\mathrm{a}\mathrm{r}\mathrm{t}}-{F}_{\mathrm{e}\mathrm{n}\mathrm{d}}\right|=\frac{4}{3}\frac{E}{1-{v}^{2}}\sqrt{R}{\delta }^{\tfrac{3}{2}} \;, $

5 $ \delta =\frac{{r}_{\mathrm{l}\mathrm{o}\mathrm{a}\mathrm{d}\mathrm{i}\mathrm{n}\mathrm{g}}t}{{k}_{p}}-\frac{F}{{k}_{p}}\; , $

where the $ {r}_{\mathrm{l}\mathrm{o}\mathrm{a}\mathrm{d}\mathrm{i}\mathrm{n}\mathrm{g}} $ denotes the pre-set force loading rate (pN/s) with which the piezo is controlled to move, $ t $ denotes the time spent from the contact starting point to end point, $ {k}_{p} $ is the spring constant of the RBC (Fig. 5C). The indentation of the droplet can be calculated as the difference between the set deformation and the real deformation of the RBC as the Eq. 5. By acquiring the $ F $ and $ t $ value and the set spring constant and force loading rate, we can obtain the Young’s modulus *E*.

## MATERIAL, EQUIPMENT AND SOFTWARE

### Buffers, reagents and other supplies

1 Coating buffer. Prepare 500 mL 0.1 mol/L bicarbonate (NaHCO_3_), 100 mL 0.1 mol/L carbonate (Na_2_CO_3_) buffer and titrate the carbonate stock solution into bicarbonate stock to yield pH 8.5.

2 N2 buffer. Dissolve 10.387 g KCl, 1.191 g NaCl, 0.067 g KH_2_PO_4_, 0.353 g Na_2_HPO_4_ and 4.852 g sucrose with 500 mL deionized water and adjust the pH to 7.2.

3 Nystatin solution. Nystatin was dissolved in DMSO in a final concentration of 5 mg/mL and stored at –80 °C.

4 Biotin-PEG-SGA (Jenkem Technology, stored at –20 °C).

5 Streptavidin modified bead (SA bead) (Spherotech, stored at 4 °C). The SA bead can also be prepared and functionalized in Lab, the procedures for glass bead silanization and functionalization have been described in detail (Chen* et al.* 2015; Ju 2019).

6 Glass/borosilicate capillary tube with outer diameter 1.0 mm and inner diameter 0.7 mm, 90 mm long.

7 Distilled water.

8 10% bovine serum albumin (BSA).

9 Purified protein sample which can form LLPS droplets.

10 Mineral oil (M8410, Sigma-Aldrich).

11 Coverslip (40 mm × 22 mm, Menzel-Glaser).

12 Microinjector (MF34G-5, World precision Instruments).

13 Glass cutter.

### Equipments

1 Home-built Biomembrane Force Probe (with necessary modules including microscope, cameras, and hydraulic/pneumatic system to provide pressure for sample aspiration, as described in the previous section).

2 Pipette puller (P-1000, Sutter Instrument).

3 Microforge (MF-900, Narishige).

4 Laboratory Vortex (Any brands).

5 Laboratory centrifuge (Any brands).

### Softwares

1 LabVIEW (National Instruments, USA).

2 User-written LabVIEW program for BFP control and operation.

3 MATLAB (MathWorks, USA).

4 User-written MATLAB program which can read and analyze experimental data.

5 Origin (OriginLab, USA).

## EXPERIMENTAL PROCEDURE

### Force probe (human RBCs) preparation

1 The RBCs were isolated from a blood drop of about 20 μL by finger prick, washed with 1 mL coating buffer after vortexing the mixture and centrifuged at 1000 r/min, 1 min for three times.

2 The washed RBCs were resuspended in 0.5 mL coating buffer containing Biotin-PEG-SGA in a final concentration of 2 mg/mL and then rotate the tube for 30 min at room temperature to achieve effective biotinylation.

3 After biotinylation, the RBCs were washed with N2 buffer three times, and resuspended the RBCs in the 0.2 mL N2 buffer containing Nystatin in a final concentration gradient from 5–20 μg/mL and rotated the tubes on ice for 1 h. This treatment adjusted the osmolarity of RBCs, the osmolarity condition of the LLPS droplet should be considered.

4 The Nystatin-treated RBCs were washed three times in N2 buffer and stored in N2 buffer containing 0.5% BSA at 4 °C.

### LLPS droplet preparation

1 LLPS Droplets are prepared in the corresponding buffer. About 3 μL phase-separated droplets were required in each experiment, and then a single droplet can be picked up for elasticity measurement. Some phase-separated droplets undergo aging after formation, so freshly made droplets are recommended.

2 To ensure measurement accuracy, the RBC must be used in an iso-osmotic solution. So, the osmolarity of RBC should be adjusted to adapt the phase separation buffer.

3 For some phase separation systems, the droplets may dissolve in protein-free buffer, thus protein solution with critical/maturation concentration should be used as chamber buffer.

### Micropipettes preparation

1 Make raw micropipettes (closed end) from borosilicate capillary tube with micropipette puller, two raw micropipettes could be made from one borosilicate capillary. The tube is heated in the middle and then pulled apart. Each raw micropipette is about half the length of the borosilicate capillary tube. The tip of each raw micropipette is closed.

2 Open the raw micropipettes at the tip using the microforge. At first, electrically heat and melt the glass sphere on the forge, then insert the tip of a raw micropipette into the melted glass sphere. Stop heating and wait for the glass sphere to cool down, the tip of the raw micropipette will be broken. Then, heat and melt the glass sphere for the second time, and then insert the broken tip of the micropipette into the melted glass sphere again, the melted glass liquid will flow into the micropipette. Watch carefully and stop as soon as the glass liquid reached the location with the desired diameter. Wait for the glass sphere to cool down and then move the micropipette away, the micropipette will be broken at that desired location, resulting in a micropipette with an open tip of the desired diameter.

3 Make at least three glass micropipettes with varied inner diameters in order to hold different experimental components: RBC, SA bead and the LLPS droplet. It is better to make more micropipettes for each type in case of micropipette is broken during the experiment. For RBC and LLPS droplets of ~10–15 μm, optimal the inner diameter of the micropipette tip is 2–4 μm. For a bead which is smaller, the inner diameter should be 1.5 μm or smaller.

### Preparation of the experimental chamber

1 Cut a coverslip into two slices and attach each of them onto one side of the metal plates on the chamber holder (Fig. 2B). The coverslip slices and the holder form a chamber with the remaining two opposite sides open, allowing the insertion of micropipettes.

2 Either the selected buffer which can introduce phase separation or protein solution which already forms phase separation can be injected into the chamber, preferably at the middle and the two open sides can be sealed with mineral oil to avoid water evaporation (Fig. 2B). Choose the buffer or protein solution based on the specific requirements of the droplet.

3 Inject the RBCs and SA beads onto separated locations of the chamber, the locations should be away from each other to prevent simultaneous attachment between the RBC and the beads.

### Micromanipulation and equipment setting

1 Place the chamber holder onto the microscope stage above the objective of BFP.

2 Fill the three micropipettes with experimental buffer by a microinjector. Attach each filled micropipette to the corresponding micropipette holder and mount the micropipette holder on the corresponding micromanipulator. Place the tip of three micropipettes into the buffer or protein solution.

3 Turn on the microscope, the piezo controller and the computer.

4 Open the sample viewer software to monitor micropipettes and experimental samples through the computer screen which was convenient for the micro-manipulation under the microscope.

5 Move all the micropipette tips into the middle of the view.

6 The zero-suction pressure of the probe micropipette which holds the RBC should be firstly characterized by adjusting the height of the water manometer tower linked with the micropipette. Mark the zero-pressure height and lower the height to produce the suction pressure to hold the RBC. Adjust the suction pressure of the other two micropipettes (target and helper) to grab a bead and a droplet as well.

7 By micromanipulation, attach the SA bead onto RBC via biotin-streptavidin ligation. The centers of the probe micropipette, the RBC and the bead should be on the same axis. Adjust the position of the target micropipette to make the center of the droplet also on the same axis of the probe system.

8 Open user-written LabVIEW software and measure the inner radius of the micropipette tip (*R*_p_), the radius of the RBC (*R*_0_) and the radius of the contacting interface between the RBC and SA-bead (*R*_c_) from the image of the whole view field. The size of the droplet can also be measured. To achieve the required spring constant of RBC, we can adjust the suction pressure ∆*p* by adjusting the height of the water manometer tower.

9 Set the operation parameters which include approaching rate ($ {r}_{\mathrm{a}\mathrm{p}\mathrm{p}\mathrm{r}\mathrm{o}\mathrm{a}\mathrm{c}\mathrm{h}\mathrm{i}\mathrm{n}\mathrm{g}} $), loading rate ($ {r}_{\mathrm{l}\mathrm{o}\mathrm{a}\mathrm{d}\mathrm{i}\mathrm{n}\mathrm{g}} $)and retracting rate ($ {r}_{\mathrm{r}\mathrm{e}\mathrm{t}\mathrm{r}\mathrm{a}\mathrm{c}\mathrm{t}\mathrm{i}\mathrm{n}\mathrm{g}} $), impinge force ($ {F}_{\mathrm{i}\mathrm{m}\mathrm{p}\mathrm{i}\mathrm{n}\mathrm{g}\mathrm{e}} $), RBC spring constant ($ {k}_{p} $), depending on the experimental needs.

### Data acquisition

1 The piezo movement was controlled by the user-written LabVIEW program. Under the control, the piezo drives the forward/backward movement of the target micropipette which holds the droplet to complete repeated cycles of approaching to, pressing and retracting from the bead according to the set parameters. as shown in Fig. 5A.

**Figure 6:**
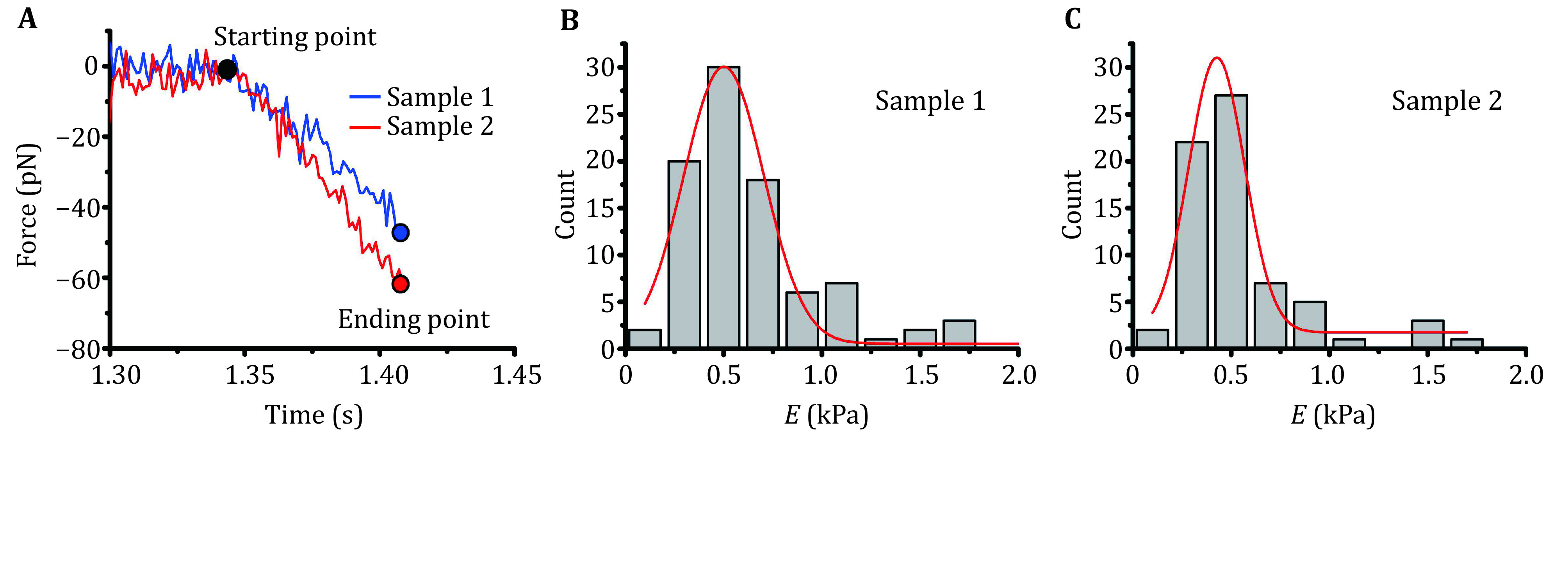
Young’s modulus of LLPS droplets measured with BFP. **A** Representative force curves of two LLPS droplet samples measured with BFP. The Young’s modulus of the droplets can be calculated by using the starting and ending points indicated on the curves. For each sample, hundreds of such measurements will be performed. **B**, **C** Histogram of the Young’s modulus of the softer Sample 1 (B) and the harder Sample 2 (C), Gaussian distribution fitting (solid red curve) gives the optimal Young’s modulus of the samples, ~0.5 kPa and ~4.8 kPa respectively

2 Adjust the piezo position during the repeated cycles to avoid the droplet moving out of the impinging axis. Hundreds of force-time curves (Fig. 6A) should be generated and stored in local disks.

### Data processing

1 Owing to the considerable amount of force curves generated from numerous experimental cycles, these curves were analyzed using user-written code of MATLAB in conjunction with LabVIEW.

　(A) A single file which contains 100 curves was firstly split into the single data file.

　(B) Read the split data file individually and repeatedly with MATLAB, determine the starting and ending point and calculate the force difference and time difference between these two points in each curve.

　(C) Using the two-point method as shown in Eq. 4 and Eq. 5 which need force difference $ F $ and time difference $ t $ between the start point of contact and the end point of the pressing process. Based on Eq. 3, the Young’s modulus$ E $ was calculated.

2 The Young’s modulus of an LLPS droplet sample from different measurements differs, and histograms could be generated for results analyzed from hundreds of measurements (Fig. 6B and 6C).

3 Origin was used to perform the Gaussian distribution fitting to statistically obtain the mean and variation (Fig. 6B and 6C). The Young’s modulus for the two samples differed. Sample 1 (~0.5 kPa, Fig. 6B) is much softer than Sample 2 (~4.8 kPa, Fig. 5C).

## Conflict of interest

Min Sun, Hui Chen, Qinghua Ji, Jianhui Xiao, Yanzhe Hou and Jizhong Lou declare that they have no conflict of interest.
